# Clinical and radiographic outcomes of pulpectomy in primary teeth using two rotary file systems compared with manual files: a cost-effectiveness analysis

**DOI:** 10.1186/s12903-025-06508-y

**Published:** 2025-07-04

**Authors:** Yasmine Ahmed Mortada Abd El fatah, Ahmad Abdel Hamid Elheeny, Mennat Allah Ashraf Abd-Elsabour, Nagwa Mohamed Ali Khattab

**Affiliations:** 1 Pediatric Dentistry and Dental Public Health Department, Faculty of Dentistry, Assiut University, Assiut, Egypt; 2 Pediatric and Community Dentistry, Faculty of Dentistry, Minia University, El Minia, Egypt; 3 Pediatric & Community Dentistry Department, Faculty of Dentistry, Ahram Canadian University, Giza, Egypt; 4 Pediatric Dentistry and Dental Public Health, Faculty of Dentistry, Ain Shams University, Cairo, Egypt

**Keywords:** Primary molars, Rotary files, Clinical success, Radiographic success, Working time, Cost-effectiveness

## Abstract

**Objectives:**

This study aimed to evaluate the clinical and radiographic success of endodontic treatment in primary molars, in addition to the evaluation of the working time (WT) and cost-effectiveness Ratio (CER), and Incremental Cost-Effectiveness Ratio (ICER) of the Fanta AF Baby and Zuanba file systems, compared to manual K-files.

**Materials and methods:**

One hundred and sixty-two-second primary molars were randomly assigned into three groups, according to the type of the instrumentation file system. A pulpectomy procedure was performed, and the average WT was recorded for each group. Clinical assessments were made at 3-, 6-, and 12-month follow-ups, while radiographic assessments were performed at 6- and 12-month follow-ups. The direct medical cost for one molar in each group was calculated, and the (CER) was determined for each group. ICER was calculated for each group, based on the average WT and radiographic success at the 12-month mark as clinical effectiveness parameters.

**Results:**

The average WT in the manual K-file group was 14.65 (± 1.85) minutes, while the Fanta AF baby file system and Zuanba file system group had an average WT of 10.52 (± 1.13) minutes, and 9.46 (± 1.12) minutes, respectively. By the end of the follow-up period, all groups exhibited the same clinical success rate of 96.7%, with no statistically significant difference (*p* > 0.05). For the radiographic success, the K-file group displayed a higher frequency of failures (11.1%), followed by the Fanta AF baby file system group (7.4%). The Zuanba file system group had the fewest failures, reporting only two cases (3.7%). Regarding ICER, the Fanta AF Baby files system was found to incur an additional cost of 50.63 EGP for reducing one minute of working time, and 52 EGP for achieving one extra successfully treated second primary molar compared to the K-files system. While the Zuanba file system costs an additional 10.98 EGP for reducing one minute of WT, and an extra 28.5 EGP for getting an extra second primary molar successfully treated compared to the K-files.

**Conclusion:**

Both assessed rotary file systems showed shorter average WT, higher radiographic success, and CER compared to the manual k file.

**Clinical relevance:**

This study aids the pediatric dentist in the choice of the most effective, both clinically and economically, rotary filling system for endodontic treatment of primary molars.

**Trial registration:**

The clinical trial was registered at clinical trial.gov identifier NCT04279041, on 2020-02-18.

## Background

There are several reasons for a dental pulp to become inflamed or infected, but by far the most common one is as a sequel to dental caries. Dental caries in a primary tooth progresses rapidly through the relatively thin enamel and penetrates dentine towards the pulp tissue, consequently, leading to pulp inflammation, infection, and loss of vitality [1, 2].

Preserving a healthy primary tooth is a critical aspect of the child’s development and quality of life [3, 4]. This is due to the important role of the primary teeth in a child’s psychological, and physical growth [3]. According to the American Academy of Pediatric Dentistry (AAPD), pulpectomy can be used to treat primary teeth with a diagnosis of pulp necrosis, furcation involvement, and apical periodontitis/ periapical abscess with no visible resorption beyond one-third of root length, allowing the molar to function until its shedding [5]. The pulpectomy procedure is complex, involving multiple steps, and requires the use of many armamentariums [2].

One of these challenges is represented in the required procedure time. The time spent during the child’s dental visit is preferred to be as short as possible to suit the child’s short attention span [2, 6]. Another factor may be the choice of root canal preparation instruments. Many choices are available in the market, which makes it difficult to choose [7, 8]. Manual files are one of the choices, with well-established evidence of their clinical effectiveness and proven clinical and radiographic success in the pulpectomy procedure for the primary molars [2, 6]. Unfortunately, their usage requires hand-driven power, which makes the pulpectomy procedure lengthy and time-consuming, exhausting both the operator and the child. Various NiTi file systems have recently been developed and are specially designed for Pediatric patients. Recent studies [2, 8–10] addressed the differences in clinical performance and success of the different types of NiTi file systems compared to the conventional manual files and concluded that the NiTi files provided a shorter instrumentation time, higher filling quality, in addition to a comparable success rate to the manual files. The main limitation facing the usage of NiTi files in pulpectomy procedures for children is their higher cost, compared to the manual files [8].

The assessment of the cost of health interventions concerning their health outcome became a critical aspect in dentists’ and patients’ everyday decision-making [11]. This is of special importance in low to meddle socioeconomic level countries, in which the individuals need to carefully consider where to spend every monetary unit to get the most possible benefit. Cost analysis measures in health care aim to assess whether the payment invested in certain techniques or equipment is worthy or not [11]. Cost analysis in the field of root canal treatment (RCT) of permanent teeth was addressed repeatedly [12–15]. These previous studies aimed to compare different RCT options in terms of their cost-effectiveness ratio (CER), to facilitate the decision-making process. Merchan et al. [14] conducted an economic evaluation study to assess the Incremental Cost Effectiveness Ratio (ICER) of three different endodontic root canal preparation techniques and concluded that the usage of the reciprocating filing technique could have an improved economic impact, in addition to the reduction in the procedure time. In the field of RCT for deciduous teeth, studies addressing economic evaluation are scarce, with only one study [13] reporting that the usage of the non-instrumentation technique involving triple antibiotic past is more cost-effective than the conventional pulpectomy procedure, in treating pulp-involved primary maxillary incisors.

To the best of our knowledge, the economic analysis of different file systems that are used in the pulpectomy procedure of the primary molars remains a knowledge gap. Also, the studies address both the Fanta AF Baby (Fanta Dental, China) and the Zuanba (Zuanba, China) file systems are barely found [16, 17]. Thus, the aim of this study was to evaluate the clinical and radiographic outcomes of pulpectomy procedures performed in primary teeth using two different rotary file systems in comparison to traditional manual files. Additionally, this study aimed to analyze the cost-effectiveness of each file system concerning radiographic success and procedural working time.

## Materials and methods

### Trial design

The current study was designed as a prospective, parallel, three-armed, randomized clinical trial, with an allocation ratio of 1:1:1. The trial arms were:

Group 1: Manual file Group (MF), the control group in which the allocated second primary molars received a pulpectomy procedure using the manual K-files (Mani Inc., Tochigi, Japan).

Group 2: Fanta AF Baby Group (FAF), in which the allocated second primary molars received a pulpectomy procedure using the Fanta AF Baby system (Fanta Dental, China).

Group 3: Zuanba Group (Z), in which the allocated second primary molars received a pulpectomy procedure using the Zuanba file system (Zuanba, China).

The trial design and reporting were run with respect to both the Consolidated Standards of Reporting Trials (CONSORT) [18], and Consolidated Health Economic Evaluation Reporting Standards (CEERS) [19].

### Ethical regulations and trial registry

The Ethical Committee of the Faculty of Dentistry, Minia University, Egypt, assessed the research protocol of the present study and approved it (reference number 360/2019). The clinical trial was registered at clinical trial.gov identifier NCT04279041, on 2020-02-18.

The study protocol followed the guidelines provided by the World Medical Association’s Declaration of Helsinki on Ethical Principles for Medical Research. The procedures and the possible discomforts or risks versus benefits were explained to the subjects and their parents/guardians, and informed consent was obtained before the initiation of the study.

### Participants

#### The inclusion criteria for the participants were

· Healthy children aged from four to seven years.

· Children who were categorized as Class I according to the American Society of Anaesthesiologists (ASA).

· Necrotic second mandibular primary molars which may be asymptomatic or manifested with dull ache pain were included.

· Percussion sensitivity or swelling close to the involved tooth accompanied with or without fistula.

· Radiographically, molars with at least two-thirds of their roots intact.

#### Exclusion criteria

· Unrestorable teeth and teeth with calcific metamorphosis inside root canals were excluded.

· Children with uncooperative behavior (Frankel rating scale I, II).

· Children whose parents refused to participate in the study or refused to sign the consent form were excluded.

· Pathological tooth mobility.

· Radiographically, internal or external root resorption, or extended radiolucency at the furcation area exceeding half of the space between the furcation and the permanent successor.

**Fig. 1:**
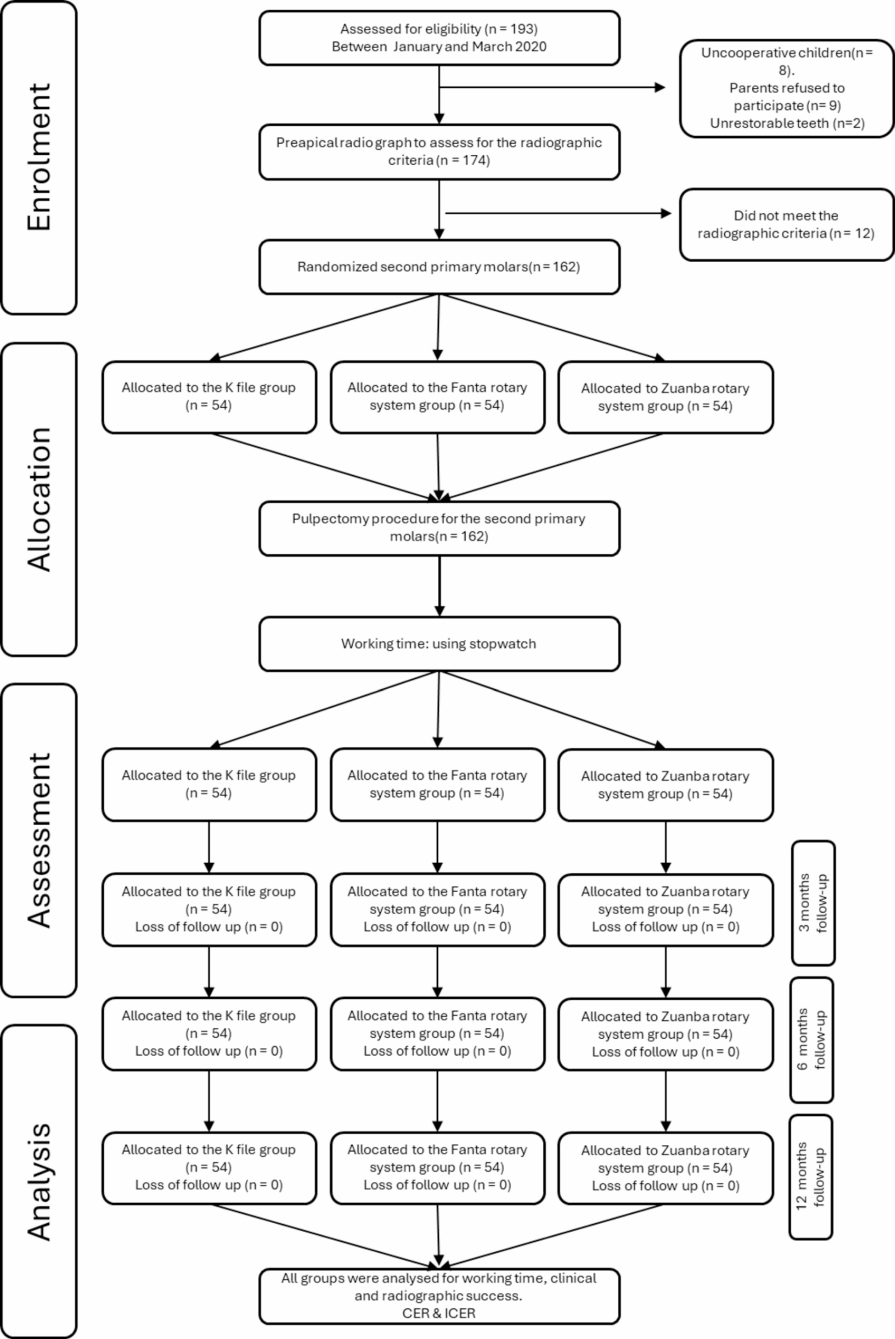
CONSORT flow chart illustrating the workflow throughout the study

· Each subject was randomized in the trial for only one molar. Figure 1 illustrates the workflow of the study.

#### Interventions and clinical procedure

The pulpectomy procedure was carried out in a single visit by the main investigator (YAMA) who is experienced using both rotary and manual instrumentation techniques. Routine nonpharmacological behavior management techniques were used throughout the procedure. Then, topical (20% benzocaine, Lolite, Dharma, Miami, USA) and local anesthesia (4% articaine hydrochloride with 1:100,000 epinephrine) were administered.

After the subjective and objective signs of the local anesthesia were achieved, rubber dam isolation was performed. Then the decayed tooth structure was excavated, and access was gained by #8 round bur at high speed. After straight-line access was obtained, pulp tissue was extirpated from the root canal using H-files. A #10 K file was then used to access the patency of the root canal. The working length was determined by superimposing an endodontic file over the preoperative radiograph and keeping it 1 mm shorter than the radiographic apex. Then, a WL confirmatory radiograph was taken.

For Group 1 (MF), the root canals were instrumented in a step-back approach up to size 35 K-file using a balanced force technique. For groups 2 (FAF), and 3 (Z) the root canal instrumentation was performed according to the recommended sequence by the manufacturer.

All rotary files were rotated with a handpiece powered by an X Smart Plus endomotor (Dentsply Maillefer, Ballaigues, Switzerland). A picking motion without pressure was applied during the mechanical instrumentation of the two rotary systems.

Root canals in the rotary file groups were prepared by adopting a crown-down technique. For all groups, the files were lubricated with 17% EDTA gel (Dolo^®^, Prevest DenPro, India). Between each file, root canals were irrigated with 5 mL of 1% sodium hypochlorite. Irrigation was performed using a 30 G side vented needle (Endo-Top; Cerkamed, Stalowa Wola, Poland). The irrigation needle was calibrated to stop 2 mm from the working length, with back-and-forth movements of 2–3 mm. Each root canal was flushed with 5 mL of normal saline as final irrigation and then dried with sterile paper points.

Root canals in all three groups were then filled with an iodoform-based calcium hydroxide paste (Metapex, Meta Biomed Co. L td Chungbuk, Korea) utilizing the pressure syringe with a plastic needle. The coronal surplus of the paste was excavated, and the coronal cavity was filled with polymer-reinforced ZOE cement (Zinconol, Prevest Denpro Limited Co, India). Finally, a preformed stainless steel crown (Kids crown, shinhung L td., Seoul, Korea) was adapted and cemented with glass ionomer cement (Micron, Prevest Denpro Limited Co, India) in the same visit.

## Outcomes

### Working time (WT)

Working Time (WT) was measured using a stopwatch that was set to start immediately after the application of the rubber dam and stopped immediately before the removal of the rubber dam. This measurement was obtained by a qualified trained dental assistant, blinded to the type of intervention.

### *Clinical and* radiographic *outcomes*

Clinical evaluations were performed at 3-, 6-, and 12-month postoperatively by other investigators (NMAK) and (AAHE) who were blinded to the instrumentation technique used in each group, while the radiographic evaluations were carried out at 6- and 12-months.

The inter- and intra-examiner reliability was tested and a Kappa score of 0.88, and 0.91were obtained, respectively.

**Fig. 2:**
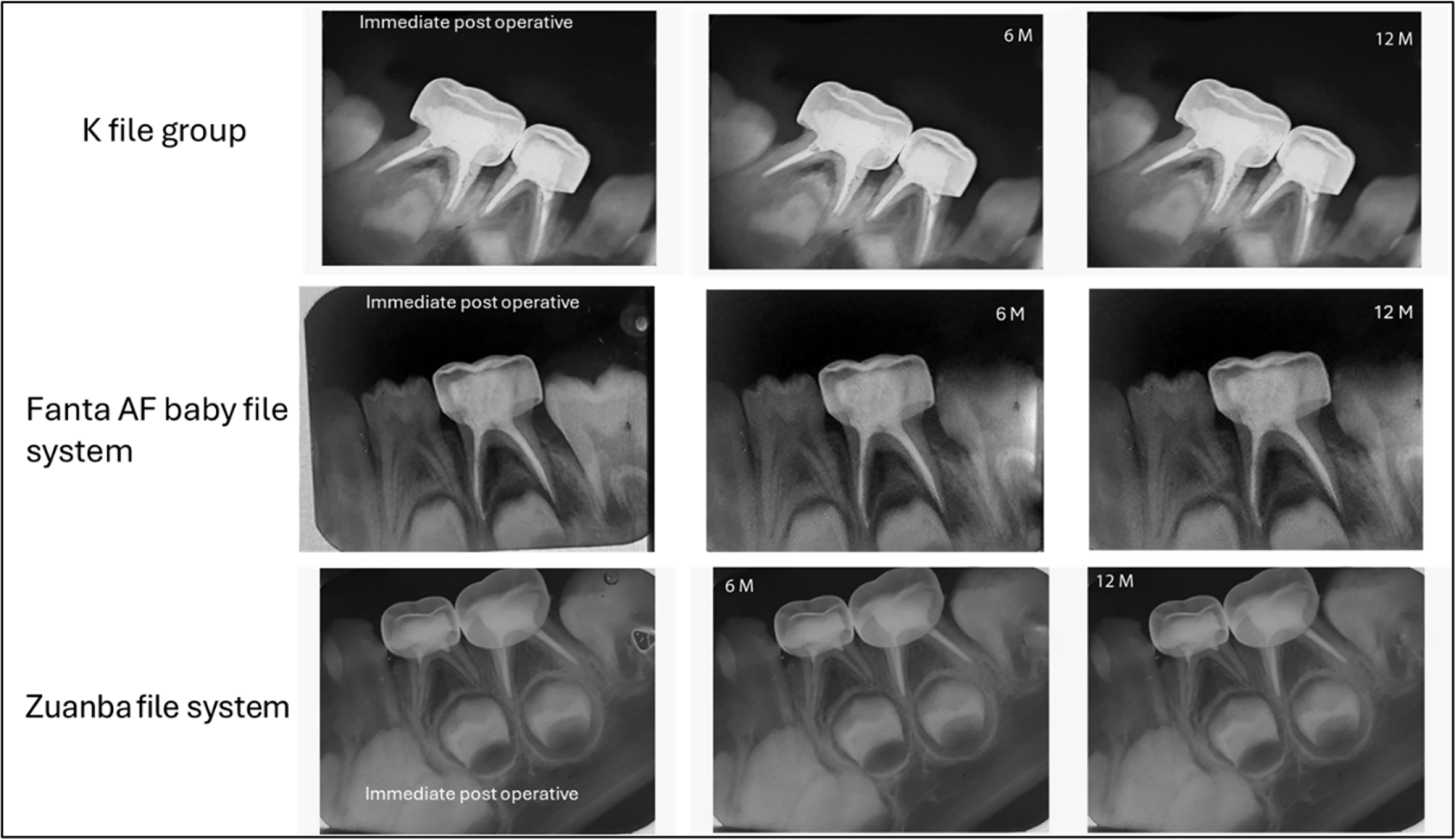
Radiographs of second primary molars treated with K-files, Fanta AF file system, or Zuanba file system at immediate post-operative, 6-month, and 12-month intervals

The criteria of successful treatment, according to which the clinical evaluation was done, were “No abnormal mobility”, “No sensitivity to percussion”, and “No swelling”. For the radiographic success, the evaluation criteria were “Preoperative pathologic inter-radicular and/or periapical radiolucency started to resolve, or remained the same size”, “No new postoperative pathological radiolucency developed”, and “No pathological internal or external root resorption”, the presence of any of these criteria was considered as failure [20]. Figure 2.

### Cost analysis

This study adopted a full economic evaluation concept, in which the cost-effectiveness analysis model was utilized. The full economic evaluation concept requires the consideration of the cost analysis of the intervention(s) in relation to a certain control [11]. In this study, the usage of the K-file system was considered as the control group.

The dentist’s (provider’s) perspective, meaning that all calculations were made to assume the cost of the provider (dentist), instead of the payer (patient) was used in this study. One-year time horizon (follow-up) was considered. There was no discounting rate applied to either the cost or the clinical effectiveness, as the implemented time horizon was less than three years [12].

Working time (WT) along with radiographic success rate at 12-month follow-up were adopted as the health effectiveness measures. While the cost estimation was done through the bottom-up (micro-costing) approach. This approach identifies the categories of the utilized resources, measures the quantities of the used resources, and then evaluates the consumed resources in the monetary unit. The consumed materials and instruments prices were obtained from manuals and sites of dental and medical sales markets in Egypt. The cost valuation for items of a permanent nature, such as endo motor handpiece, was made by dilution of their initial cost according to the manufacturer’s life span recommendations and based on the assumption that the average number of patients (molars) treated per day is eight patients.

Only direct medical cost was considered because any other cost, such as the cost of illness, productivity loss cost, or transportation cost, was expected to be equal among the participants in the three groups. Due to the nature of the current study, only the items, instruments, materials, or equipment that differed among the three groups were considered in the cost estimation, because any other costs, such as the cost of human resources, were assumed to be leveled among the three groups. The average direct medical cost of the three groups was calculated and utilized in the cost analysis. Patients with failed pulpectomy procedure treatment were retreated with the same correspondent intervention filling system, and the additional cost of their retreatment was considered in the cost analysis model. All costs were calculated in Egyptian pound (EGP) currency (1 United States Dollar equals 47.96 EGP, on 6/5/2024).

The cost-effectiveness ratio (CER) for each of the three study groups was calculated using the following equation:

$$\:CER\hspace{0.17em}=\hspace{0.17em}C\:/\:E$$

where C is the average cost calculated for the group, and E is the health effectiveness measure for the same group (WT, or radiographic success rate at 12-month- follow-up).

Incremental cost-effectiveness ratio (ICER) was calculated for the two intervention groups, in relation to the control group using the following equation:

$$\:ICER\:=\:(CI\:-\:CC)\:/\:(EI\:-\:EC)$$

where C_I_ is the average cost of the intervention group (Fanta AF Baby or Zuanba file systems), C_C_ is the average cost in the control group, E_I_ is the health effectiveness measure (WT, or radiographic success rate at one-year follow-up) in the intervention group (Fanta AF Baby or Zuanba file systems), and E_C_ is the health effectiveness measure in the control group.

### Sample size estimation

Number of primary second molars per group was calculated according to Charan and Biswas [21], following the equation: sample size = 2(Zα/2 + Zβ) 2 P(1-P) / (P1-P2)2. Where Zα/2 = Z0.05/2 = Z0.025 = 1.96 (From Z table) at type 1 error of 5% and Zβ = Z0.20 = 0.842 (From Z table) at 80% power. While P1 − P2 = Difference in proportion of events in two groups P = Pooled prevalence = [prevalence in case group (p1) + prevalence in control group (p2)]/2. Accordingly, the sample size was determined 47 primary second molars per group. To compensate for possible dropout, 15% of the calculated sample size was added, so that the final sample size was 162 primary second molars (54 per group).

### Randomization and allocation concealment

The current study utilized a convenient consecutive sampling technique. Randomization of the used filing system was achieved by using the block randomization technique with opaque sealed envelopes concealment technique. Sequence generation, using computer-generated sequence (https://www.sealedenvelope.com/simple-randomiser/v1/lists), and allocation concealment, using sealed envelopes, were done by an independent researcher who was not involved in the study. The implementation of the participants into the corresponding study group was done using serially numbered concealed envelopes opened by the investigator (MAA) just after working length determination.

### Blinding

This trial was double-blinded, in which the outcome assessors (NMAK) (AAHE) who assessed the clinical and radiographic success, and the research assistant who measured the WT, in addition to the data statisticians were blinded. The blinding was not possible neither for the main investigator (YAAMA) nor for the participants, due to the nature of the study. The manual files are easily distinguished from the rotary ones, and the two rotary systems had different colors and sizes.

### Statistical methods

Statistical analysis was done using SPSS 20 ®, and Microsoft Excel 356 ®. The qualitative data was presented as frequencies and percentages and were compared using the Chi-square test. While the quantitative data was presented as mean and standard deviation (SD) and were compared using the one-way ANOVA test followed by Bonferroni’s Post Hoc test for multiple comparisons, after confirming its normality using Kolmogorov–Smirnov. The p-value significance level was set at 0.05.

## Results

Out of 193 patients assessed for eligibility, 162 met the criteria and were randomized among the study’s three arms (*n* = 54 per group). All subjects presented for follow-up at all periods. The baseline characteristics of each group’s participants are shown in Table 1. The recruitment took place during the period between January to March 2022. All assigned participants were analyzed according to the group they were first randomized to.

Evaluation of the average WT revealed that the average WT in the k file group was 14.65 (± 1.85) minutes, while in the Fanta AF baby files and Zuanba File system group were 10.52 (± 1.13) minutes, and 9.46 (± 1.12) minutes, respectively. The mean costs for treating one second primary molar in each group were found to be 74, 282, and 131 EGP in the k file group, Fanta AF baby file system group, and Zuanba file system group, respectively. WT and costs of the study’s three groups are reported in Table 2.

No statistically significant difference between the three groups over the follow-up periods (*p* > 0.05) was observed regarding the clinical success, Table 3. After 3 months the clinical success rates in the three groups were 100%. At 6 months of the follow-up period, the success rate of the K-file groups and the Fanta AF baby file system group was 96.3% where two cases (3.7%) were reported as clinical failures in each group, due to the presence of pain on percussion. In the Zuanba file system group, the success rate was 100% at 6 months follow-up. By the end of the follow-up period, all groups reported the same clinical success rate (96.3%). Two failures were observed in the Zuanba file system group, one due to the presence of tooth mobility and the other due to the presence of pain on percussion. No statistically significant difference between groups throughout the follow-up periods (*p* > 0.05).

For radiographic success, Table 4, shows no statistically significant difference between the three groups over the follow-up periods (*p* > 0.05). At 6 months, the success rate of the two rotary file groups was 96.3%, while four cases (7.4%) were reported as failures in the K-file group. After 12 months, the k file group displayed a higher frequency of failures (6 cases 11.1%), followed by the Fanta AF baby file system group (4 cases 7.4%), and finally, Zuanba file system group which reported only two failures cases (3.7%). All of the failed cases showed an enlargement of the existing apical radiolucency or developed a new radiolucency.

No adverse effects related to the study interventions were observed.

The cost-effectiveness ratio (CER), and (ICER) of WT and radiographic success at 12 months follow-up are demonstrated in Table 5 and 6, respectively. Fanta AF baby file system would cost an extra 50.63 EGP, and 52 EGP for reducing one minute of WT, and obtaining an extra second primary molar successfully treated compared to the k files system, respectively. While Zuanba file system was found to cost an extra 10.98 EGP, and 28.5 EGP for reducing one minute of WT, and obtaining an extra second primary molar successfully treated compared to the K-files system, respectively.

**Table 1:** Baseline characteristics among the study groups

Baselines	MF group	FAF group	Z group	*P* - value
Gender Boys N(%) Girls N(%)	30(55.6) 24(44.4)	35(64.8) 19(35.2)	33(61.1) 21(38.9)	0.612
Age Mean (SD)	4.96 ± 0.70	4.93 ± 0.75	4.70 ± 0.79	0.153

Chi-square test for the gender and One-way ANOVA for the mean age, p-value set to ≤ 0.05

**Table 2:** Average working time (minutes) and the direct medical cost per second primary molar (by Egyptian pound) of the study groups

	MF group	FAF group	Z group	*P* - value	
Average working time Mean (SD)	14.65a (1.85)	10.52b (1.13)	9.46c (1.12)	< 00001*	
Average direct medical cost (Egyptian pound)	74	282	131	------------	

* One-way ANOVA test with Bonferroni post hoc

Counts with different superscript letters were significantly different (Bonferroni post hoc)

**Table 3:** Clinical success and failure among all groups at 3, 6, and 12 months

Follow up period	Clinical success/failure	MF group *N*(%)	FAF group *N*(%)	Z group *N*(%)	*p*-value
3 months	Success Failure	54(100) 0(0)	54(100) 0(0)	54(100) 0(0)	N/A
6 months	Success Failure	52(96.3) 2(3.7)	52(96.3) 2(3.7)	54(100) 0(0)	0.359
12 months	Success Failure	52(96.3) 2(3.7)	52(96.3) 2(3.7)	52(96.3) 2(3.7)	0.591

Chi-square test, p-value set to ≤ 0.05

**Table 4:** Radiographic success and failure among the study groups at 6, and 12 months

Follow up period	Radiographic success/failure	MF group *N*(%)	FAF group *N*(%)	Z group *N*(%)	*p*-value
6 months	Success Failure	50(92.6) 4(7.4)	52(96.3) 2(3.7)	52(96.3) 2(3.7)	0.591
12 months	Success Failure	48(88.9) 6(11.1)	52(96.3) 2(3.7)	50(92.6) 4(7.4)	0.340

Chi-square test, p-value set to ≤ 0.05

**Table 5:** Cost-effectiveness and incremental cost-effectiveness ratios according to the average working time among all/ or study groups

Groups	Cost-effectiveness ratio (CER) (Egyptian pound/minute)	Incremental cost-effectiveness ratio (ICER) (Egyptian pound/minute)
MF group	5.05	Reference
FAF group	26.80	− 50.36
Z group	13.84	− 10.98

**Table 6:** Cost-effectiveness and incremental cost-effectiveness ratios according to the radiographic success at 12-month follow-up among the study groups

Groups	Cost-effectiveness ratio (CER) (Egyptian pound/ success rate)	Incremental cost-effectiveness ratio (ICER) (Egyptian pound/ extra successfully treated molar)
MF group	84.09	Reference
FAF group	293.75	52
Z group	141.62	28.5

## Discussion

Pulpectomy procedure in children is an everyday practice of the pediatric dentist. The pediatric dentist found himself challenged by the wide range of available instrumentation systems in the market to choose from. The current study was conducted to illustrate both the success and cost-effectiveness of two of the available pediatric rotary systems in the market and highlight the differences between them and the conventional manual K-file system.

Second primary molars were only treated in this study as their anatomical features with deep occlusal fissures make them more prone to dental caries, also, they are usually more accessible for practical work as well as their radiographic interpretation is much easier without superimposition which enables the investigators to identify any radiographic change. In addition, the success rate of the pulpectomy procedure was found to be higher in the second primary molars when compared to the first primary molars [22].

All treatment procedures were carried out by a single operator, and the root canal preparation was done in accordance with the manufacturer’s instructions, and according to the AAPD recommendations to eliminate performance bias [5]. The outcomes assessment was done by a neutral dental assistant in the case of WT determination, or blinded investigators for the clinical and radiographic evaluation, to avoid detection bias. Also, the study followed a well-designed adherent protocol in order to avoid attrition bias.

Metapex was used as root canal-filling material in all three groups. Calcium hydroxide-iodoform paste (Metapex) gives excellent results when used as a filling material in pulpectomy and is available premixed in polypropylene syringes, which reduces the WT [23].

To our knowledge, this is the first randomized controlled clinical trial to report the application of both pediatric files (Fanta AF baby and Zuanba) in pulpectomy of primary teeth. Therefore, there is no similar literature to compare with the present results. Thus, the overall outcome was compared with that observed in earlier studies using other file systems in primary teeth.

The result of the current study showed that after 12-month follow-up, 96.3% of teeth in both manual and rotary groups were clinically asymptomatic. This finding goes in accordance with several previous studies carried out by Babu & Kavyashree, who also reported 100% clinical success [24]. Also, it agrees with both Manchanda et al. [6] and Chugh et al. [2] who concluded in their systematic reviews and meta-analyses that there is no difference between rotary files and manual files regarding clinical success.

However, these results contradicted those obtained by Morankar et al. [10] as they reported a high degree of clinical success for pulpectomy procedures in primary molars performed with manual instrumentation (92.3%) compared to the rotary group (85.2%). This variation in clinical success rates between both studies could be attributed to the difference in the study population, eligibility criteria, treatment protocol (irrigants and obturating materials), or the longer follow-up period, which was up to 24-month.

The reported radiographic success by the end of the follow-up (12-month) was 88.9%, 96.3%, and 92.6% for the K-file, Fanta AF baby file system, and Zuanba file system groups respectively. Despite, the rotary groups showed higher performance than the manual group, no statistically significant differences were detected among the three groups. This finding is consistent with the results of Babu and Kavyashree [24]. However, the results disagree with the findings of Amorim et al. [25] who explained radiographic failures observed in the experimental group due to the accumulation or overflow of dentin shavings in the periapical region, which was further exacerbated by the rhizolysis of primary teeth.

This discrepancy between clinical and radiographic success reported in the present study could be attributed to silent radiographic failures which occurred in six teeth treated with the manual technique and in four teeth treated with the Zuanba rotary technique. These teeth added to the radiographic failure though they were clinically asymptomatic [10].

The average WT in the current study was found to be less in the file groups, with a statistically significant difference. This result is in accordance with Hadwa et al. [16] who investigated the instrumentation time in the Fanta AF baby file system and Kedo-S square rotary system compared to the manual k file system. Also, it goes in accordance with Jeevanandan & Govindaraju [26] who investigated the same outcome, comparing the Kedo-S square file system with the manual k files, and got the same conclusion. It is undeniable that the usage of the rotary files would reduce the WT, which is an important requirement for young children.

To our knowledge, this is the first study to assess the cost-effectiveness and incremental cost-effectiveness ratio of the pediatric rotary files. The cost valuation for each of the study groups, cost-effectiveness ratio, and incremental cost ratio were carried out as per Merchan et al. [14]. The results of the cost analysis revealed that for only an extra 50.36 EGP, or 52 EGP, a reduction of the WT by one minute, or extra primary molar successfully treated could obtained if the Fanta AF baby files system was used instead of the manual k files, respectively. For the Zuanba file system, the extra cost would be 10.98 EGP, or 28 EGP, for the same gain. This illustrated the high cost-effectiveness of the rotary files over the manual files. Also, it should be noted that the usage of the rotary files provides other superior qualities over the manual files that are not accounted for in the current economic model, such as the quality of obturation, reduced operator fatigue, reduced probability of canal transportation, and reduced post-operative pain.

This study is one of the very limited RCTs that shed light on Fanta and Zuanba file systems in the RCT of primary molars with a representative sample and sufficient statistical power. Also, it is the first study to assess the cost-effectiveness of rotary files for deciduous molars compared to manual files. On the other hand, the main challenges were the relatively short follow-up period (12-month), participants could be followed for a longer period to evaluate clinical and radiographic outcomes of different instrumentation systems. Second, the use of 2D conventional radiographic imaging to evaluate radiographic success. The digital radiograph or CBCT would be a possible better alternative, however, ethical concern hinders this choice. Also, the absence of data regarding the number of patients undergoing pulpectomy procedures each year, and the data about the willingness to pay in Egypt, limited the assumptions of the cost analysis model for the study population.

The applicability of the current study results among other populations is subject to some factors, health condition of the child, the expertise of the operator, and the financial aspects of the population. Although, the main results of this study, regarding its outcomes, are expected to remain valid among other populations. Moreover, the applicability of this study’s results in other countries is dependent on several economic factors related to these countries, although the proportional economic analysis results of this study are expected to be maintained.

## Conclusion

Both rotary file systems showed superior average WT, radiographic success, and more cost-effectiveness compared to the manual K-files. It is recommended that the pulpectomy procedure in the primary molars be performed using rotary files.

## Data Availability

Raw data (master table) is available upon request from the corresponding author.
